# Mental health and psychosocial support for South Sudanese refugees in northern Uganda: a needs and resource assessment

**DOI:** 10.1186/s13031-016-0085-6

**Published:** 2016-09-07

**Authors:** Alex Adaku, James Okello, Blakeley Lowry, Jeremy C. Kane, Stephen Alderman, Seggane Musisi, Wietse A. Tol

**Affiliations:** 1 Peter C. Alderman Foundation Uganda, Arua, Uganda; 2 Peter C. Alderman Foundation Uganda, Gulu, Uganda; 3 Peter C. Alderman Foundation, Bedford, New York USA; 4 Department of Mental Health, Johns Hopkins Bloomberg School of Public Health, Baltimore, MD USA; 5 Peter C. Alderman Foundation Uganda, Mawanda Road, Plot 855, 1st floor, Kampala, Uganda; 6 Department of Psychiatry, Makerere University, Kampala, Uganda

## Abstract

**Background:**

Since December 2013, an armed conflict in South Sudan has resulted in the displacement of over 2.2 million people, more than 270,000 of whom are presently in refugee settlements located throughout Uganda. Existing literature suggests that refugees are at increased risk for a range of mental health and psychosocial problems. There is international consensus on the importance of needs and resource assessments to inform potential mental health and psychosocial support (MHPSS) interventions.

**Methods:**

We conducted a MHPSS needs and resource assessment in Rhino Camp refugee settlement in northern Uganda, between June and August 2014. We followed World Health Organization (WHO) and United Nations High Commissioner for Refugees (UNHCR) guidelines for MHPSS needs assessments in humanitarian settings. The assessment used a range of methodologies including: 1) a desk (literature) review to understand the context for mental health service provision; 2) an analysis of data from existing health information systems (HIS); 3) an assessment of the current infrastructure for service provision using a shortened version of a *Who does What Where until When (4Ws)*; and 4) semi-structured individual and group interviews (total *n* = 86) with key informants (*n* = 13) and general community members (individual interviews *n* = 28, four focus groups with *n* = 45).

**Results:**

Data from the HIS indicated that visits to health centers in refugee settlements attributable to psychotic disorders, severe emotional disorders, and other psychological complaints increased following the refugee influx between 2013 and 2014, but overall help-seeking from health centers was low compared to estimates from epidemiological studies. In semi-structured interviews the three highest ranked mental health and psychosocial problems included “overthinking”, ethnic conflict, and child abuse. Other concerns included family separation, drug abuse, poverty, and unaccompanied minors. The 4Ws assessment revealed that there were very limited MHPSS services available in Rhino Camp.

**Conclusions:**

The types of MHPSS problems among South Sudanese refugees in northern Uganda are diverse and the burden appears to be considerable, yet there are currently few available services. The assessment indicates the need for a range of services addressing social concerns as well as varied types of mental conditions. The idiom of “overthinking” may form a useful starting point for intervention development and mental health communication.

## Background

Armed conflict continues to be a major concern for populations around the globe. In 2014, 40 armed conflicts were recorded, together associated with the highest conflict yearly fatality rate since the Cold War [1]. According to the United Nations High Commissioner for Refugees (UNHCR), there are an increasing number of refugees around the world, with an estimated 13 million in 2014 [2]. The majority of refugees reside in low- and middle-income countries (LMIC).

Armed conflict and conflict-related displacement have psychological and social consequences for individuals, families, and communities. Displacement can lead to disruptions of traditional family and community structures that support mental health [3]. Although successful adaptation to adverse circumstances occurs for most people (e.g. through employing positive coping strategies to target stressors or their psychological consequences), others may develop mental health disorders and pre-existing mental illness may go untreated [4, 5]. Mental disorders in conflict-affected populations have been shown to be associated with significant impairment in functioning and productivity [6–8]. Addressing the mental health and well-being of populations exposed to armed conflict should therefore be treated as a priority.

A meta-analysis by Steel and colleagues [9] of epidemiological studies among conflict-affected populations found a point prevalence of 17.3 % for depression and 15.4 % for post-traumatic stress disorder (PTSD) in a subset of the most rigorous studies, suggesting that these are considerable problems. However, limitations in the existing epidemiological literature make it challenging to build on such studies as a basis for intervention planning. First, a combination of methodological (e.g., sampling problems, use of measures that do not distinguish between normal psychological distress and mental disorder, and lack of cultural validation) and contextual factors (e.g., not accounting for pre-existing mental health conditions) have contributed to a very high variability in prevalence rates [10]. Second, most epidemiological studies with conflict-affected populations, and refugees in particular, are focused on symptoms of common mental disorders following exposure to potentially traumatic events, such as depression, PTSD, and anxiety, whereas it is acknowledged that a much wider range of mental health concerns affects refugees. For example, a systematic review found that “thinking too much” is a common idiom of distress that has variable overlap with common mental disorders, but also suggests aspects of experience, distress, and social positioning not captured by psychiatric diagnoses [11]. In addition, pre-existing neuropsychiatric disorders such as psychosis and epilepsy are important for mental health service planning for conflict-affected populations [12, 13]. Beyond knowledge from epidemiological studies, further information is necessary for the design of contextually appropriate MHPSS interventions.

There is consensus on the importance of mental health and psychosocial support needs and resource assessments. The Inter-Agency Standing Committee (IASC) Guidelines on Mental Health and Psychosocial Support in Emergency Settings [14], developed by a group of 27 United Nations and non-governmental organization (NGO) humanitarian agencies, state that assessments should be part of the ‘minimum response’ to humanitarian crises. These guidelines recommend the following topics for inclusion in needs and resource assessments: relevant demographic and contextual information; experience of the emergency; mental health and psychosocial problems; existing sources of psychosocial well-being and mental health; organizational capacities and activities; and programming needs and opportunities [14].

These guidelines, however, do not provide details on what types of methodologies may be applied to collect the required information. In response, the WHO and UNHCR developed a toolkit for MHPSS needs and resource assessments [15]. Recommended assessment methods in this document include: a review of published and grey literature; collecting existing information from relevant stakeholders; gathering new information through integrating questions related to psychosocial and mental health concerns in assessments from diverse sectors and; addressing knowledge gaps by collecting new information on mental health and psychosocial issues through interviews or surveys [15]. In this paper, we describe an application of the WHO-UNHCR toolkit to assess mental health needs and resources with South Sudanese refugees in northern Uganda.

### Conflict in South Sudan

In 2011, the Republic of South Sudan became an independent nation after decades of civil war. Continued conflicts with Sudan and internal instability have plagued the nation since independence. In December 2013, a power struggle between President Kiir and his ex-deputy Riek Machar broke out over an alleged coup. Subsequent fighting has occurred along ethnic lines (Dinka and Nuer) and has resulted in thousands of people killed and 2.1 million people displaced, the majority inside South Sudan [16]. As of January 2016, over 644,000 refugees have fled into the neighboring countries of Ethiopia, Kenya, Sudan, and Uganda [17]. On August 26, 2015, the conflicting parties signed a peace agreement, but fighting has continued.

Uganda had previously received a significant number of South Sudanese refugees, and refugee settlements in northern Uganda have experienced a rapid influx of refugees displaced by the recent violence. UNHCR estimates that there are more than 290,000 refugees (from before and after December 2013) located in refugee settlements in Adjumani, Arua, Kiryandongo and Koboko districts [18].

### Current assessment

The assessment was implemented by the Peter C. Alderman Foundation (PCAF), an organization that works with public partners (e.g. governments and public universities) to establish mental health and psychosocial support services in post-conflict countries. Since 2005, PCAF has provided services through a partnership with the Ministry of Health and Makerere University in Uganda [19]. A PCAF multi-disciplinary team has been located in the Arua Regional Referral Hospital in northern Uganda since 2010 and consists of a supervising psychiatrist, psychiatric clinical officer, social worker, counselor, nurse, and a community social worker liaising with community health workers. The team makes bi-weekly visits to the Rhino Camp settlement and conducts regular health education sessions and psychoeducation on various topics during the outreaches. As a result of the recent political violence in South Sudan, thousands of refugees have flooded into Uganda and have set up residence in several sites, including in Rhino Camp and surrounding settlements. PCAF was interested in knowing what types of additional services it is placed to offer for South Sudanese refugees in the West Nile region. The goal of the assessment was to better understand the mental health and psychosocial needs and resources of the South Sudanese refugees living in the Rhino Camp settlement in Arua in order to develop recommendations for PCAF MHPSS interventions in the settlement.

## Methods

### Setting

The Rhino Camp refugee settlement is located in Arua district, which is part of the West Nile sub-region of northwestern Uganda, bordered by the Democratic Republic of Congo (DRC) to the west and South Sudan to the north. Rhino Camp was the first refugee settlement in the West Nile Region, established in the early 1960s [20]. The specific area was selected because of land availability, proximity of the area to refugees’ countries of origins such as southern Sudan and the DRC, and ethnic similarity to host populations [21]. UNHCR manages the Rhino Camp in collaboration with the Ugandan government’s Office of the Prime Minister. As of December 2015, there were a recorded 22,520 South Sudanese refugees in the Rhino Camp. The population is approximately 48 % male and 52 % female [22].

### Selection of assessment tools

The assessment applied methods from a WHO-UNHCR (2012) toolkit on mental health and psychosocial needs and resource assessments in humanitarian settings [15]. The toolkit recommends that, to start with, a selection from among 12 tools is made to tailor assessments to each unique humanitarian context and the phase of the conflict. For example, in the immediate first days after a major humanitarian crisis it may not be possible to complete rapid ethnographic research in a timely enough way to inform MHPSS response. In this phase, a rapid assessment of basic survival and protection needs of people with mental disorders living in institutions may be more helpful. Use of qualitative methods may be more helpful the first month or so after a major crisis, when the situation on the ground becomes less fluid.

Selection of tools for the current assessment was driven by existing gaps in information to assess suitability of current mental health services and needs for potential service expansion. With these gaps in mind, the assessment started with the development of assessment objectives collaboratively by the PCAF teams in Arua and Kampala. We then chose appropriate tools from the WHO-UNHCR guide to carry out each objective. The objectives and corresponding tools for the needs assessment are summarized in Table 1. Broadly, we employed two types of assessment activities to meet the assessment objectives: 1) building on existing information; and 2) primary data collection.

**Table 1:** Needs assessment objectives and tools

Assessment objective	WHO-UNHCR assessment tool applied
What is the context for mental health service provision?	Checklist for getting information from other agencies Template for desk review
What is the mental health burden in the refugee population?	Review of PCAF’s Health Information System (client documentation) and UNHCR Health Information System
What are perceived mental health and psychosocial support needs of key stakeholders?	Free listing activity with general community members
What are critical social and cultural considerations in the provision of mental health services?	Template for desk review
What is the current infrastructure for provision of mental health and psychosocial support services?	Shortened version of Who does What Where until When (4Ws) Assessment of formal mental health resources (interviews with key informants) Checklist for getting information from other agencies

### Building on existing information

The WHO-UNHCR toolkit recommends that assessments build on existing information, in order to avoid duplication of data collection, and to reduce the burden on assessment participants and wastage of valuable humanitarian resources [15]. We built on existing information through three methods: 1) a desk review; 2) an analysis of existing health information systems (PCAF’s existing patient database and UNHCR’s Health Information System data); and 3) a rapid 4 W’s (Who is doing What, Where until When) assessment.

The desk review was completed by compiling data reports, peer-reviewed literature, and grey literature found from ReliefWeb, UNHCR, WHO, World Bank, Makerere University, NGOs, doctoral theses, and local newspapers. PubMed was also searched, using combinations of the following search terms: “mental health”, “Sudanese refugees”, “psychosocial”, “Arua”, “West Nile”, “Rhino Camp”, “northern Uganda”, and “Sudan”. Relevant articles were identified from the references of the peer-reviewed journal articles resulting from the PubMed search.

UNHCR’s Health Information System (HIS; also referred to as Twine-- http://twine.unhcr.org/app/) has been active in five refugee settlement in Uganda since 2009, including the Rhino Camp refugee settlement. The HIS is a surveillance system within the refugee camps that captures the number of visits to primary care centers for the following types of mental, neurological, and substance use (MNS) disorders: epilepsy, alcohol/substance, mental retardation, psychotic disorder, severe emotional disorder (i.e., severe symptoms of sadness and anxiety associated with marked impairment in functioning), somatic complaint and ‘other psychological’ (psychological symptoms without marked impairment in functioning). We extracted the number of raw visits for each MNS category and present the proportion of visits attributable to each of the seven MNS categories (c.f. [12]).

As a final step in our review of existing information on the current context of psychosocial services in Rhino Camp, we conducted a rapid 4 W assessment as recommended by the WHO-UNHCR toolkit (cf. [23]). We contacted each of the agencies engaged in MHPSS activities in Rhino Camp to learn more about what their specific objectives and outreach efforts were, where those efforts were concentrated, and for how long the activities were planned to continue. We created a list of agencies, their MHPSS activities, and contact information. We then assigned MHPSS activity codes to each agency based on criteria developed by the IASC Reference Group for Mental Health and Psychosocial Support in Emergencies [15].

### Primary data collection: semi-structured interviews and focus groups

In order to rapidly capture local perspectives on mental health, psychosocial problems, and coping strategies, we collected qualitative data from two participant types: key informants and general community members. The key informant interviews were held with thirteen people selected because of their leadership role in the settlement and their expected strong knowledge on mental health and psychosocial needs and resources in the settlement: three local council leaders, five refugee leaders, two aid agency workers, and three female health workers. The interviews focused on which types of formal (governmental) mental health services are available and to compile an overview of which types of non-governmental agencies were implementing different types of services (see below).

We also conducted semi-structured interviews and focus groups with general community members in the settlement, selected through convenience and purposive sampling. We purposively selected locations to ensure that we would be interviewing mainly newly arrived refugees, as compared to refugees residing in Rhino Camp before the new hostilities. We conducted interviews in Tika village in zone B and in Ocea in zone A. Ocea is the initial reception center for Rhino Camp, and Tika has larger numbers of new arrivals. In both places we contacted refugee leaders to discuss our interest in conducting interviews. We subsequently moved around in the area recommended by the refugee leader as having new arrivals, and knocked on random huts to start the consent process with available individuals. Individual interviews were conducted with twenty-eight adults and we convened four focus groups: one women’s group with twelve members; one men’s group of seven members; one girls’ group of fifteen members (aged 14 to 22 years), and one boys’ group of eleven members (aged 16 to 21 years). The individual interviews focused on free listing. We first asked an open question to participants about the general problems faced in the settlement, and to provide a brief description of each problem. We probed for four potential MHPSS problem areas: problems with thoughts, feelings, social relationships and behavior. Later group interviews with general community members focused on mental health and psychosocial concerns mentioned in response to the general question in the free listing interviews. We asked the focus group participants to rank the selected mental health and psychosocial problems in terms of their priority. We also asked participants to discuss how the prioritized mental health and psychosocial problems may impact daily activities, and methods refugees apply to cope with the prioritized mental health and psychosocial problems.

Orientation and training on the data collection tools took place in June 2014 with assessment participant interviews occurring one month later. Training to the interviewers was provided over a 3-day period by two psychiatrists from the region (AA and JO). Participants’ backgrounds were as follows: one psychiatric clinical officer; one psychologist; one social worker; one nurse; and two graduate community psychology students on an internship with the organization. The training focused on interviewing skills and ethical interviewing practice, both through didactic training and ample opportunity for practice through role-playing interviews. Training materials consisted of the WHO-UNHCR assessment toolkit, and the interview guides.

Interviews were conducted in English and Juba Arabic and translated into English by community leaders and mobilizers. All research procedures were conducted in line with the Helsinki declaration, but we did not submit a protocol for formal ethical review given that participants were not asked about their personal experiences but were asked in general about their opinions on priorities for mental health and psychosocial support interventions in Rhino Camp, and we did not anticipate any risk in participation. We did seek permission from the Rhino Camp commander (Office of the Prime Minister, Uganda); the UNHCR in Arua; the Ministry of Health District Health Officer of Arua; and the Chairperson of the Community Welfare Committee and zone leaders. All officials agreed on the importance of the initiative and did not anticipate any risks with participation. All participants were informed about the nature of the interviews by the interviewers, and were asked for their written consent/assent to be interviewed. Interviewed adolescents provided their own consent for interviewing. The collected information was kept secure and all efforts were made to protect participants’ anonymity.

The qualitative data from semi-structured interviews and focus groups were analyzed using straightforward content analysis techniques. The formats of the interviews (available upon request) were structured to facilitate more rapid analysis as opposed to e.g. development of a grounded theory, in order to provide timely information. For easy collation, interview formats provided boxes for notes after each question, rather than capturing verbatim text. We copied and pasted in to summary tables all notes made in response to the same question across participants. In these summary tables we grouped together responses that were similar in nature, and gave these groups ‘codes’. These codes form the basis of the results described below.

## Results and discussion

### Desk review

The desk review gathered information from over 80 documents including data reports, peer-reviewed literature, and grey literature found from Relief Web, UNHCR, WHO, World Bank, Makerere University, NGOs, doctoral theses, and local newspapers. The desk review briefly summarized information from the identified documents on: the geographical context; socio-demographic characteristics; historical context; political and legal context; religious context; economic situation and education; gender and family aspects; cultural considerations; general health; and mental health and psychosocial wellbeing specifically. Of note is that the review indicated a policy of “self-reliance” promoted by the Ugandan government, in which refugees are provided a plot of land and allowed to settle in government-appointed areas. In these areas, according to government policy, refugees are permitted to receive government-funded regional health and educational resources. Despite this generous policy, our review found that South Sudanese refugees have reported a lack of access to basic needs and health care services.

With regard to mental health specifically, the desk review identified both social science research and psychiatric epidemiological studies. The papers were limited in scope and we did not identify research that has been published focused on refugees displaced in the current armed conflict. The psychiatric epidemiological research on populations displaced from South Sudan in previous conflicts suggested high prevalence of common mental disorders (depression, PTSD, anxiety), although the use of non-validated cut-off scores for the population may mean that psychological distress and mental disorders are conflated in these studies [24]. Exact expected rates of mental disorders are challenging to estimate from existing studies. Nevertheless, based on these studies it may reasonably be expected that similarly high levels of psychological distress are present in South Sudanese refugees currently displaced. In line with this research and general estimates from the WHO, it may be estimated that a large percentage of people experience psychological distress; 15 % to 20 % may experience mild or moderate common mental disorders (for example mild and moderate forms of depression, anxiety disorders, PTSD); and 3 % to 4 % may experience severe mental disorders such as psychosis, severe depression, severely disabling form of anxiety disorder, PTSD) [15]. The desk review also points to the importance of attention to sexual and other forms of gender-based violence (SGBV) as a risk factor for mental health concerns. Reports from earlier periods of displacement point to early marriages being a key issue, and current reports also highlight SGBV during flight and resettlement as key concerns.

The qualitative research included in the desk review examined insider’s (emic) perspectives on ill mental health. Recent qualitative studies [25, 26] point to mental health problems as very broadly being viewed as ‘madness’ (severe mental disturbances associated with aggressive and bizarre behavior) or ‘sadness’ (difficulties associated with psychosocial causes such as loss, bereavement and deprivation). The problem of ‘overthinking’ or ‘thinking too much’ is also repeatedly mentioned in qualitative studies. Earlier qualitative research with southern Sudanese displaced populations [27, 28] similarly notes the importance of ‘thinking too much’, and suggests that psychological distress may be considered as a problem in and of itself. ‘Thinking too much’ has been reported as an important idiom of distress in multiple populations, typically referring to ruminative, intrusive, and anxious thoughts. It has been found to have overlap with common mental disorders such as depression, anxiety, and PTSD, but should not be interpreted as a gloss for psychiatric disorder as it captures aspects of experience, distress, and social positioning not captured by psychiatric diagnoses [11].

Overall, the qualitative literature describes how the self in displaced South Sudanese populations has not commonly been reported as a separate entity, but that the self, identity and the body are seen as inextricably linked. Metaphors concerning the body are used to describe psychological distress (e.g. heart pain, bad heart) [27, 28]. Communal coping strategies (social support through connecting with elders, relatives, community members, religious institutes) are consistently mentioned in both earlier and later qualitative research as coping and help-seeking strategies [27, 28].

### UNHCR health information system data

Data from the Health Information System includes the number of visits to primary health care centers in the refugee settlement for mental, neurological, and substance use problems across seven categories. These data revealed that visits attributable to psychotic disorder, severe emotional disorder, and other psychological complaints in Adjumani, Kiryandongo, and Kyangwali (other districts where South Sudanese refugees are settled) increased from 2013 to 2014 following the start of the refugee influx. In Kiryandongo (average population of 3,944 refugees), where data were collected over 35 months from March 2010 to January 2014, 4.8 % of visits were for psychotic disorder, 1.4 % for severe emotional disorder, and 0.7 % for other psychological complaints. Whereas for the six months data collected during February to July 2014 (and where the average population had grown considerably to an average of 24,973 per month), 9.6 % of the visits were for psychotic disorder, 2.4 % for severe emotional disorder and 3.6 % for other psychological. In Rhino Camp during January through July 2014 (average population of 16,875 per month), HIS data indicated that 4.2 % of visits were for psychotic disorder, 16.7 % for severe emotional disorder, and 25 % of visits classified as other psychological [29].

It is unlikely that these increased numbers represent the high percentage of the population that is potentially affected by mental health and psychosocial problems. As qualitative studies identified by the desk review indicated, displaced South Sudanese have been more likely to seek health care if a physical problem is suspected to be underlying mental health issues.

### Clinical data from Peter C. Alderman Foundation

Data collected between 2013 and 2014 at both the health facilities in the refugee settlement and PCAF’s clinic based in the regional referral hospital also indicates an increase in the burden of mental health problems. In 2013, the total number of clients seen was 339, of which 264 (78 %) were seen during outreach visits. According to the 2014 annual patient data report, the Arua clinic treated increased to 389 patients, of which 314 (87 %) were seen during community outreaches. Visits to health centers also increased between 2013 and 2014 among youth, particularly males: 93 boys under 18 were enrolled in services in 2014 compared to 71 in 2013.

### 4Ws assessment

The rapid 4Ws shows that there is very limited mental health and psychosocial support available in the Rhino Camp. A limited number of child protection actors have focused on implementing child-friendly spaces; providing information about SGBV; referring children to health and education services; providing legal support to SGBV survivors; building community capacity in child protection issues; and family tracing and reunification.

In addition, more focused mental health and psychosocial support has been provided by the Uganda Red Cross Society, Medical Teams International (MTI) and PCAF. The Red Cross provided psychological first aid between March and July 2014. MTI is providing structured recreational activities and has a psychosocial officer. PCAF conducts weekly outreaches through the four lower level health centers with a multi-disciplinary team located at the Arua Regional Referral Hospital.

A summary of findings from the desk review, Health Information System, PCAF clinic data, and 4Ws assessment is included in Table 2.

**Table 2:** Summary of key findings from review of existing information

Desk review	Refugees report lack of access to basic needs despite Uganda government's resettlement policy;
	Attention should be paid to SGBV, which may be highly prevalent during earlier periods of displacement;
	High levels of psychological symptoms in populations displaced from South Sudan prior to the current crisis;
	Qualitative findings highlight the importance of ‘overthinking’;
	Communal coping strategies (e.g., family social supports) are perceived to be helpful
Clinic data	Clinic visits for psychotic disorders, severe emotional disorders, and ‘other psychological complaints’ increased following refugee influx and patient data indicates an increase in burden of mental health problems
4Ws	Very limited mental health and psychosocial support available in the Rhino Camp; Child protection actors have been: implementing child-friendly spaces, providing information about SGBV, referring children to health and education services, providing legal support to SGBV survivors, building community capacity in child protection issues, and conducting family tracing and reunification Uganda Red Cross Society, MTI and PCAF have provided MHPSS

### Semi-structured interviews and focus group discussions

In response to the question on general concerns of refugees in Rhino Camp settlement, interview and focus group participants (characteristics described in Table 3) often indicated that a lack of access to sufficient food, water, and shelter in the settlement as well as infectious diseases and lack of general health care services were pressing needs. It is notable that “overthinking” was the second most common concern mentioned by participants in response to the general question on overall problems faced by refugees in Rhino Camp. Furthermore, although not viewed as a specific mental health or psychosocial problem by community members, the anxiety associated with inadequate food distribution and the resulting hunger can be seen as concerns with important psychological consequences and critical to take into account when developing MHPSS interventions [14].

**Table 3:** Overview of assessment participants in primary qualitative data collected

Key Informant Interviews (*n* = 13)	General community members (*n* = 73)
Local council leaders (*n* = 3)	Individual interviews with general community members (*n* = 28)
Refugee leaders (*n* = 5)	4 focus group discussions (*n* = 45): with women (*n* = 12); with men (*n* = 7); with school-aged girls (*n* = 15); with school-aged boys (*n* = 11)
Aid agency workers (*n* = 2)	
Health services workers (*n* = 3)	

As noted, participants mentioned mental health and psychosocial concerns in response to the question on general problems in Rhino Camp. We selected these problems for further discussion in subsequent group interviews, and asked participants to prioritize these concerns. The three most highly prioritized mental health and psychosocial problems by participants in focus groups were: “overthinking” (mentioned in individual interviews by more than half of the participants), ethnic conflicts (mentioned in individual interviews by around half of the participants), and child abuse (prioritized highly across the four focus groups, although not mentioned frequently in the individual interviews). Furthermore, frequently mentioned concerns (but not part of the top three most highly prioritized concerns) included: family separation; drug abuse; poverty; and unaccompanied minors.

It is clear that the current crisis has had both important psychological impacts as well as social consequences for the South Sudanese refugees in Rhino Camp settlement. In group interviews participants emphasized that the prioritized mental health and psychosocial problems affect refugees’ daily life, as well as how families and the community function. When discussing the problem of “overthinking” in group interviews, participants mentioned that this has been triggered by being forced to flee their homes, losing family members and living in a refugee settlement, whereas ethnic conflicts are likely a result of the ethnic strife that has accompanied the current fighting in South Sudan and has been brought into the displacement settings. The trifecta of family separation, child abuse, and a poor education system were seen as critical social issues in the settlement potentially poses significant risk to the larger groups of families and children living in Rhino Camp. A summary of themes from the semi-structured individual and group interviews is presented in Table 4.

**Table 4:** Summary of themes from key informant interview and focus group discussions

Topic	Themes
General problems in refugee settlement	Lack of access to basic needs
	Disease
	Insufficient health services
	Security issues
Most important MHPSS problems	Ethnic conflict
	Overthinking
	Child abuse
Help-seeking strategies	Coping through connectedness with tribe
	Social support from family/friends
	Advice from elders and church leaders
	Community resolution of problems

Although the key informant and group interviews with community members were more exploratory in nature, findings on how participants perceive mental health and psychosocial problems echo more in-depth qualitative studies on South Sudanese refugees. The concept of “overthinking” was considered one of the most important mental health and psychosocial concerns among assessment participants. Coker [28] interviewed southern Sudanese refugees in Egypt and found that the refugees often discussed their illnesses in social or existential terms rather than in physical terms. “Thinking too much” was a common factor in illness and was thought to “fuel” their illnesses. Another qualitative study among South Sudanese showed that they attributed the causes of mental illness to their life situation (such as lack of basic needs), thinking too much, being impatient, family situation, consequences of war, and health problems [26]. As noted above, a recent systematic review has focused on ‘thinking too much’ as an important idiom of distress in multiple populations [11].

According to assessment participants, coping strategies overall (particularly for thinking too much and ethnic conflicts) centered on connectedness to a tribe, social support, seeking advice from elders and church leaders, and coming together to resolve community problems. Other suggestions to combat problems such as drug abuse and child abuse focused on education and community sensitization. Help-seeking patterns and coping strategies mentioned by interview participants were also similar to those found in past research with South Sudanese refugees. Connectedness to a tribe, social support, seeking advice from elders and church leaders and coming together to resolve community problems were reported as the main help-seeking behaviors. Regarding how individuals should receive treatment for mental illness, one qualitative study among the South Sudanese showed that participants listed social support, practical support such as help in accessing basic needs, medical care, prayer and traditional treatments, no treatment (e.g. belief that some conditions are not treatable), and government solutions (e.g. government should care for people with mental disorders) [26]. Two qualitative studies among Sudanese refugees resettled in Australia revealed that coping strategies used during their period of transit (such as in refugee camps), in the order of most commonly identified, include the use of religion, social support networks, cognitive reframing of the situation, and focusing on the future personal and political aspirations [30, 31].

### Limitations of the applied assessment techniques

There were several limitations of this needs assessment. First, based on the desk review findings, we know that limited research has been conducted with the South Sudanese population in the West Nile Region, and in the Rhino Camp specifically, so the knowledge base to draw on is limited. This includes both mental health epidemiological studies and piloting of interventions, as well as information on local mental health-related expressions, idioms and sources of distress, and help-seeking patterns. Moreover, the majority of the existing information has been gathered with refugees who have resettled in developed countries. There is also limited information on the role of the formal social sector in providing psychosocial support to the target population. More research should be conducted to explore other aspects of mental health and well-being, and how these are conceptualized by South Sudanese refugees themselves [32].

Second, the methods employed in this needs assessment are limited in scope and rigor. Our aim was to conduct an assessment that could rapidly inform decisions on service provision, rather than provide a thorough understanding of South Sudanese refugee perspectives on mental health and psychosocial wellbeing. Although the qualitative interviews sought to fill some of the gaps in understanding how South Sudanese refugees perceive and cope with mental health and psychosocial problems, it is important to note that this is an exploratory assessment and more detailed ethnographic research is recommended for a more in-depth understanding. Although the key informant and group interviews provide a feasible method for collection of information on perspectives with regard to MHPSS priorities, they yields less contextual details and generalizability of the information is uncertain. Further, we were unable to assess the quality of services provided by the few existing services at the camps.

Third, it is important to note that the information was collected by a non-governmental organization working in partnership with the Ministry of Health in Uganda. The fact that PCAF staff conducted the interviews could have biased some responses. For example, participants mentioned engaging PCAF services as coping mechanisms for mental health problems. Finally, although PCAF has been working in the Rhino Camp for several years, language and socio-cultural barriers may have led to some information being missed.

Fourth, we did not seek ethical approval for the primary data collection, because we did not consider the collected information to be human subjects research (i.e., we asked about people’s general opinions on the situation in Rhino Camp). Advantages of this approach are feasibility and timeliness of information collection, especially in settings with limited resources for internal review boards. Disadvantages of this approach are that participants did not share individual experiences and that personally sensitive topics were likely avoided.

## Conclusions

Findings from this assessment suggest that the current crisis has had important mental health and psychosocial consequences for South Sudanese refugees. By following a WHO-UNHCR toolkit [15], we feel the assessment methodology allowed for efficient data collection, combining existing and primary information that can serve as the foundation for MHPSS activity recommendations, while minimizing the burden on participants. In this sense, the assessment aimed to find a balance between relevance (i.e., ensuring timely collection of information with an eye on intervention planning) and excellence (i.e., ensuring that collected information has sufficient reliability and validity, for example by reaching data saturation and triangulating information), while trying to reduce demand on time of the displaced population [33]. At the same time, the assessment allowed for an engagement with refugees in an open-ended manner. Although necessarily limited in scope and depth, we feel a number of conclusions can be drawn from the assessment.

First, the assessment appears to support the importance of multi-layered interventions as described in international consensus-based guidelines on best practices in MHPSS [14, 34]. These guidelines refer to MHPSS services consisting of 1) social considerations in basic services and security (e.g., advocating for services that address basic needs in a safe, socially appropriate manner that protects dignity); 2) strengthening community and family supports (e.g., activating socially supportive networks at the community-level, addressing family violence); 3) focused (person-to-person) non-specialized supports (e.g., basic mental health care by primary health care workers); and 4) specialized services (e.g., mental health care by specialists such as psychiatrists, clinical psychologists, etc.) [14]. In public health terms, such interventions may be conceptualized on the continuum of mental health promotion, prevention of mental disorders, and treatment and maintenance [35, 36]. Similarly, participants in the current assessment stressed the importance of basic needs being met, despite the generous settlement policy of the Ugandan government. In addition, important social concerns were emphasized by participants, including ethnic tensions in the settlement and protection issues such as child abuse and sexual and other forms of gender-based violence. These social concerns are well-known risk factors for mental health and psychosocial wellbeing [37, 38]. Furthermore, assessment participants highly prioritized the problem of “overthinking” in semi-structured interviews, whereas the assessment of existing health information systems also showed increased help-seeking in health care centers for (pre-existing) neuro-psychiatric disorders such as psychosis and epilepsy. In conclusion, a range of services to address these diverse needs at different levels appears warranted.

Second, the assessment highlights the importance of sensitivity to contextual issues. The most important mental health and psychosocial concern according to assessment participants was “overthinking”. “Thinking too much” has frequently appeared in ethnographic studies of psychological distress with populations outside of North America and Europe and is included as a cultural concept of distress in the most recent version of the Diagnostic and Statistical Manual. A recent systematic review found that “thinking too much” has often been compared to depression, anxiety, PTSD and psychological distress more broadly, but as a term is stigmatized less. A wide range of perceived causes has been reported, including past adverse events and ongoing stressors such as economic and structural barriers to meeting basic needs. However, the review’s authors caution to equate “thinking too much” with psychiatric disorders as classified in international diagnostic systems, given the potential for idioms of distress to range from non-pathological states to disordered states, their heterogeneous presentation, and for the idiom to communicate social ruptures and processes [11]. Although this requires further exploration, participants in group interviews in this assessment emphasized the importance of currently ongoing displacement-related stressors such as a lack of access to basic needs, living away from one’s home, and bereavement. At the same time, social concerns such as ethnic tensions and (child) protection issues in the settlement were highlighted by participants. Further ethnographic research would be useful to understand the etiology, phenomenology, and sequelae of thinking too much in this context, but the idiom would seem a useful starting point in intervention development and health communication.

Third, we note the discrepancy between data on help-seeking from the health information systems and knowledge identified through the desk review on potential rates of mental disorders as identified in population-based epidemiological studies. There may be multiple reasons for this discrepancy, including: help-seeking behavior (e.g., systematic differences in the types of problems for which refugees seek assistance for mental health concerns in health care centers); recognizability (e.g., the extent to which staff in health care centers identify different types of mental health concerns); treatability and quality of care (e.g., the extent to which people will return to a health care center when they perceive it can be treated effectively there) [12]. Overall, it is challenging to estimate the prevalence of mental disorders in Rhino Camp, but there is likely a large group of people with mental concerns that impair their functioning who do not present to the health care system. At the same time, assessment participants reported that accessibility to general health care was limited, and an overview of existing MHPSS services found limited availability of services. This situation may require an alternative (inter-sectoral) strategy to address the existing mental health burden, for example through task-shifting of interventions to increase reach of supports into more remote areas in the settlement, as well as community-based prevention efforts aimed at strengthening refugees’ existing individual and communal coping efforts.

In closing, an important point to emphasize is that we identified limited MHPSS services in the settlement, despite the desk review and assessment participants identifying mental health and psychosocial concerns as critical issues. Although participants mentioned existing supports and coping strategies they can rely on to address these concerns, overall the assessment indicates largely unmet needs for services to promote and protect psychosocial wellbeing and prevent and treat mental disorders in the Rhino Camp settlement.

## Abbreviations

4Ws, Who does What Where until When; DRC, Democratic Republic of the Congo; HIS, Health Information System; IASC, Inter-Agency Standing Committee; LMIC, Low- and Middle-Income Countries; MHPSS, Mental Health and Psychosocial Support; MNS, Mental, Neurological, and Substance Use; MTI, Medical Teams International; NGO, Non-Governmental Organization; PCAF, Peter C. Alderman Foundation; PTSD, Post-Traumatic Stress Disorder; SGBV, Sexual and other forms of Gender Based Violence; UNHCR, United Nations High Commissioner for Refugees; WHO, World Health Organization
